# Human Enterovirus 68 Interferes with the Host Cell Cycle to Facilitate Viral Production

**DOI:** 10.3389/fcimb.2017.00029

**Published:** 2017-02-08

**Authors:** Zeng-yan Wang, Ting Zhong, Yue Wang, Feng-mei Song, Xiao-feng Yu, Li-ping Xing, Wen-yan Zhang, Jing-hua Yu, Shu-cheng Hua, Xiao-fang Yu

**Affiliations:** ^1^Department of Internal Medicine, The First Hospital of Jilin University, Jilin UniversityChangchun, China; ^2^Medicinal Chemistry, College of Pharmacy, Changchun University of Chinese MedicineChangchun, China; ^3^Chemistry of Traditional Chinese Medicine, College of Pharmacy, Changchun University of Chinese MedicineChangchun, China; ^4^Department of Experimental Pharmacology and Toxicology, School of Pharmacy, Jilin UnivrsityChangchun, China; ^5^Institute of Virology and AIDS Research, The First Hospital of Jilin University, Jilin UniversityChangchun, China

**Keywords:** enterovirus 68 (EV-D68), cell cycle, G0/G1 arrest, viral replication, host-pathogen interaction

## Abstract

Enterovirus D68 (EV-D68) is an emerging pathogen that recently caused a large outbreak of severe respiratory disease in the United States and other countries. Little is known about the relationship between EV-D68 virus and host cells. In this study, we assessed the effect of the host cell cycle on EV-D68 viral production, as well as the ability of EV-D68 to manipulate host cell cycle progression. The results suggest that synchronization in G0/G1 phase, but not S phase, promotes viral production, while synchronization in G2/M inhibits viral production. Both an early EV-D68 isolate and currently circulating strains of EV-D68 can manipulate the host cell cycle to arrest cells in the G0/G1 phase, thus providing favorable conditions for virus production. Cell cycle regulation by EV-D68 was associated with corresponding effects on the expression of cyclins and CDKs, which were observed at the level of the protein and/or mRNA. Furthermore, the viral non-structural protein 3D of EV-D68 prevents progression from G0/G1 to S. Interestingly, another member of the *Picornaviridae* family, EV-A71, differs from EV-D68 in that G0/G1 synchronization inhibits, rather than promotes, EV-A71 viral replication. However, these viruses are similar in that G2/M synchronization inhibits the production and activity of both viruses, which is suggestive of a common therapeutic target for both types of enterovirus. These results further clarify the pathogenic mechanisms of enteroviruses and provide a potential strategy for the treatment and prevention of EV-D68-related disease.

## Introduction

Human enterovirus 68 (EV-D68) is an emerging pathogen that can cause severe respiratory disease and is associated with cases of paralysis, especially among children. It was first isolated from samples obtained in California in 1962 from four children with pneumonia and bronchiolitis (Schieble et al., 1967). Over the past 10 years, EV-D68 infection outbreaks have been reported in Italy, the United States, Germany, China, and several other countries (Esposito et al., 2015; Farrell et al., 2015; Reiche et al., 2015; Zhang et al., 2015), with a record number of confirmed cases in 2014 (http://www.cdc.gov/non-polio-enterovirus/about/ev-d68.html). Unfortunately, no vaccines for prevention or medicines for treatment are currently available for future outbreaks, mainly due to the fact that information on host factors required for EV-D68 replication is scarce.

EV-D68 belong to enterovirus (family *Picornaviridae*, genus *Enterovirus*), which are non-enveloped, positive-sense single-strand RNA viruses of approximately 7500 nt and contain a large open reading frame that encodes a polyprotein that is cleaved to yield corresponding viral proteins. Based on the molecular and biological characteristics, four human enterovirus (HEV) species are currently designated as HEV-A, -B, -C, and -D (Oberste et al., 1999a,b). The representative of HEV-A serotype is human enterovirus 71 (EV-A71), which is a primary causative agent for Hand, foot, and mouth disease (HFMD) that is associated with the recent outbreaks in Asia (Liu et al., 2011; Wang et al., 2012). EV-D68 is assigned to HEV-D serotype, but EV-D68 is unlike other enteroviruses in that it is acid labile and biologically more similar to human rhinoviruses that are associated with respiratory diseases (Smura et al., 2010).

As a feature of their pathogenic mechanism, many viruses facilitate their own replication by interacting with host factors that regulate cell cycle progression. Examples can be discovered in DNA viruses, retroviruses and RNA viruses. DNA viruses, which replicate in the nucleus, have been extensively investigated in regard to control the cell cycle of host cells. For example, some small DNA viruses including simian virus 40 (DeCaprio et al., 1988), adenovirus (Howe et al., 1990; Eckner et al., 1994), and human papillomavirus (Werness et al., 1990), which lack their own polymerases, use the host polymerase to promote the entry of cells into S phase from G0/G1 phase. For other large DNA viruses, for example, herpesviruses can induce G0/G1 arrest in order to avoid competing for cellular DNA replication resources (Flemington, 2001). Cell cycle regulation also has been observed for retroviruses, which, like DNA viruses, replicate in the nucleus. The Vpr protein of human immunodeficiency virus type 1 is responsible for eliciting cell cycle arrest in G2/M phase (He et al., 1995; Goh et al., 1998). Furthermore, RNA viruses, whose primary site of replication is normally the cytoplasm, have also been demonstrated to interfere with the host cell cycle. Infectious bronchitis virus (IBV) induces an S and G2/M-phase arrest to favor viral replication (Dove et al., 2006; Li et al., 2007), and mouse hepatitis virus (MHV) (Chen and Makino, 2004) and some severe acute respiratory syndrome coronavirus (SARS-CoV) proteins induce cell cycle arrest in G0/G1 phase (Yuan et al., 2005, 2006). In a previous study, we found that human EV-A71 and Coxsackievirus A16, manipulate the host cell cycle at S phase in order to promote their own viral replication (Yu et al., 2015); however, the potential manipulation of the host cell cycle by EV-D68, which is associated with higher lethality in recent large-scale outbreaks, has not been previously characterized.

In the current study, we examined the effects of the cell cycle status on EV-D68 viral replication, as well as the impact of EV-D68 virus on the host cell cycle. Our data show that EV-D68 replication is integrally associated with the host cell cycle, though the pattern of regulation differs distinctly from that of EV-A71. These results further increase the understanding of the pathogenic mechanisms of enteroviruses and provide a potential target for the treatment and prevention of enterovirus-related diseases.

## Materials and methods

### Viruses and cells

The Fermon (ATCC, VR-1826), US/KY/14-18953 (ATCC, VR-1825D), and US/MO/14-18947 (ATCC, VR-1823D) strains of EV-D68; and the Changchun077 strain of EV-A71 have been reported previously (Wang et al., 2012). Viruses were propagated in human rhabdomyosarcoma RD cells (No CCL-136), and the supernatants were harvested and stored at −80°C. Human embryonic kidney cells (HEK 293T cells) (No CRL-11268) and RD cells were purchased from the ATCC (Manassas, VA, USA) and used according to a previous study (Wang et al., 2015). Cells were maintained in Dulbecco's modified Eagle's medium (DMEM) (Hyclone, Logan, UT, USA) supplemented with 10% fetal bovine serum (FBS) (GIBCO BRL, Grand Island, NY, USA).

### Viral titer determination

The viral titers were determined by measuring the 50% tissue culture infective dose (TCID50) in a microtitration assay using RD cells, as described (Gay et al., 2006). RD cells were seeded and incubated at 37°C for 24 h in 96-well plates. Virus-containing supernatant was serially diluted 10-fold, and 100 μl of diluent virus was added per well in octuplicate. Until the experimental endpoint was reached the cytopathic effect was observed once per day. According to the Reed-Muench method (Reed, 1983) the viral titers of the TCID50 were determined, based on the assumption that material with 1 × 10^5^ TCID50/ml will produce 0.7 × 10^5^ plaque forming units/ml (www.protocol-online.org/biology-forums/posts/1664.html).

### Infection

Cells were mock-infected or infected with EV-D68 or EV-A71 at a multiplicity of infection (MOI) of 0.8. After 2 h of virus adsorption, cells were washed with phosphate-buffered saline (PBS) one time, then added fresh culture medium.

### Cell cycle release

Subconfluent cultures of RD cells were synchronized in G0/G1 phase by serum deprivation (He et al., 2010). Approximately 5 × 10^5^ cells were plated in a 6-well plate and maintained in serum-free medium for 24 h. After EV-D68 virus infection, fresh 10% DMEM was added to release the cells from G0/G1.

### Synchronization of cells

In order to observe the effects of the cell cycle on virus growth, subconfluent cultures of RD cells were synchronized in G0/G1 phase by serum deprivation for 24 h (He et al., 2010). For S-phase synchronization, a final concentration of 0.85 mM thymidine (Sigma) were added (Helt and Harris, 2005; Yu et al., 2015) for 24 h. For G2/M synchronization, 25 ng/ml of nocodazole (Sigma) was added (He et al., 1995; Yu et al., 2015) for 24 h. For sustained S and G2/M cell-cycle arrest after virus infection, cells were treated with fresh 0.85 mM thymidine and 25 ng/ml nocodazole for the indicated times.

### Cell cycle analysis by flow cytometry

Propidium iodide (PI) staining was used to measure the nuclear DNA content according to previous study (Yu et al., 2015). Firstly, the cells were collected and fixed with 1 ml of cold 70% ethanol at 4°C overnight and then re-suspended in PI staining buffer (50 μg/ml PI (Sigma), 20 μg/ml RNase in PBS) for 2 h at 4°C. Fluorescence-activated cell sorting (FACScan; BD) were used to analyze the PI-stained cells, and at least 10,000 cells were counted for each sample. ModFit LT, version 2.0 (Verity Software House) was performed for data analysis.

### Western blot analysis

Virus-infected or mock-infected cells were collected at various times after EV-D68 infection and washed once with PBS as previously described (Yu et al.). The following antibodies were used in Western blot analyses: anti-CDK2 (Cell Signal), anti-cyclinE1 (Proteintech), anti-CDK4 (Cell Signal), anti-CDK6 (Cell Signal), anti-cyclinD (Cell Signal), anti-CDK1 (Boster), anti-cyclinB1 (Santa Cruz), and anti-histone (GenScript). Secondary antibodies from mouse or rabbit were obtained from Jackson Immuno Research.

### Quantitative real-time PCR

All work was carried out in a designated PCR-clean area as previously described (Yu et al.). RNA was extracted from infected and uninfected cells using Trizol reagent (Gibco-BRL, Rockville, Md.) and isolated as specified by the manufacturer. The RNA was DNAse-treated (DNase I-RNase-Free, Ambion) to remove any contaminating DNA; 200 ng of total RNA was reverse-transcribed with oligo dT primers using the High Capacity cDNA RT Kit (Applied Biosystems) in a 20 μl cDNA reaction, as specified by the manufacturer. For quantitative PCR, the template cDNA was added to a 20 μl reaction with SYBR GREEN PCR Master Mix (Applied Biosystems) and 0.2 μM of primer (Table 1). The amplification was carried out using an ABI Prism 7000 for 40 cycles under the following conditions: initial denaturation at 95°C for 10 min; 40 cycles of 95°C for 15 s and 60°C for 1 min. The fold changes were calculated relative to GAPDH using the ΔΔCt method.

**Table 1:** **The primer for real time PCR and plasmid construct**.

**Primer pair**	**Role**	**Forward sequence5′–3′ (restriction enzyme)**	**Reverse sequence5′–3′ (restriction enzyme)**
EV-D68 VP1 (Fermon)	Real time	CACCATACTCACAACTGTGGC	AATGAAATGAATCCTGCTCCT
EV-D68 VP1 (US/KY/14-18953)	Real time	GCCCTTACTCCAGAAAAACA	CAAAACCATCATAGAAAACT
EV-D68 VP1 (US/MO/14-18947)	Real time	CGTGGGTCTTCCTGACTTGA	GGGGGGTCGGAGATTTTAAA
EV-A71 VP1	Real time	AGCACCCACAGGCCAGAACACAC	ATCCCGCCCTACTGAAGAAACTA
CyclinE	Real time	TCAGGGTATCAGTGGTGCGA	CAAATCCAAGCTGTCTCTGTG
CDK2	Real time	CTCCTGGGCTCGAAATATTATTCCACAG	CCGGAAGAGCTGGTCAATCTCAGA
CDK4	Real time	AAGCCGACCAGTTGGGCAAAAT	GCTCCACGGGGCAGGGATACAT
CDK6	Real time	GGTCAGGTTGTTTGATGTGTGC	TATCCTTTATGGTTTCAGTGGG
CylcinD	Real time	CTACTACCGCCTCACACGCTTC	TCCTCCTCCTCTTCCTCCTCCT
CDK1	Real time	TCAAGTGGTAGCCATGAAAAAA	TAACCTGGAATCCTGCATAAGC
CyclinB1	Real time	TGGCCTCACAAAGCACATGA	GCTGTGCCAGCGTGCTAATC
GAPDH	Real time	GCAAATTCCATGGCACCGT	TCGCCCCACTTGATTTTGG
3D	Plasmid construct	AACTGCAGACCATGTACCCTTACGACGTCCCAGATTACGCGGGTGAGATAGTTAGCAATGAGA (PST1)	CGGGATCCCTAAAACGAATCTAACCATTTCCG (BamH1)
3C	Plasmid construct	AACTGCAGACCATGTACCCTTACGACGTCCCAGATTACGCGGGACCAGGATTTGATTTT (PST1)	CGGGATCCCTATTGTGTATCAGTAAAGTAAGAGT (BamH1)

*3D and 3C forward primer with hemagglutinin (HA) sequence: (TACCCTTACGACGTCCCAGATTACGCG)*.

### Enzyme-linked immunosorbent assays

The cell lysates were examined for CDK4, CDK6, cyclinD1, CDK2, cyclinE1, CDK1, cyclinB1 and histone with ELISA kits (Meiyan, Shanghai, China) according to the manufacturer's instructions. The microplate was quantified using a microplate reader (Bio-Rad, Hercules, CA, USA). Target protein expression was normalized to the histone expression.

### Statistical analyses

Statistical differences were analyzed using the Student's *t-*test for all analysis, except of 3C and 3D dose-dependent test in **Figures 4B,D** with Pearson correlation coefficient. Data are presented as means and standard deviations (SD). ^*^P-values of <0.05 were considered statistically significant.

## Results

### Synchronization at different cell cycle stages has profound effects on EV-D68 production

**Figure 1:**
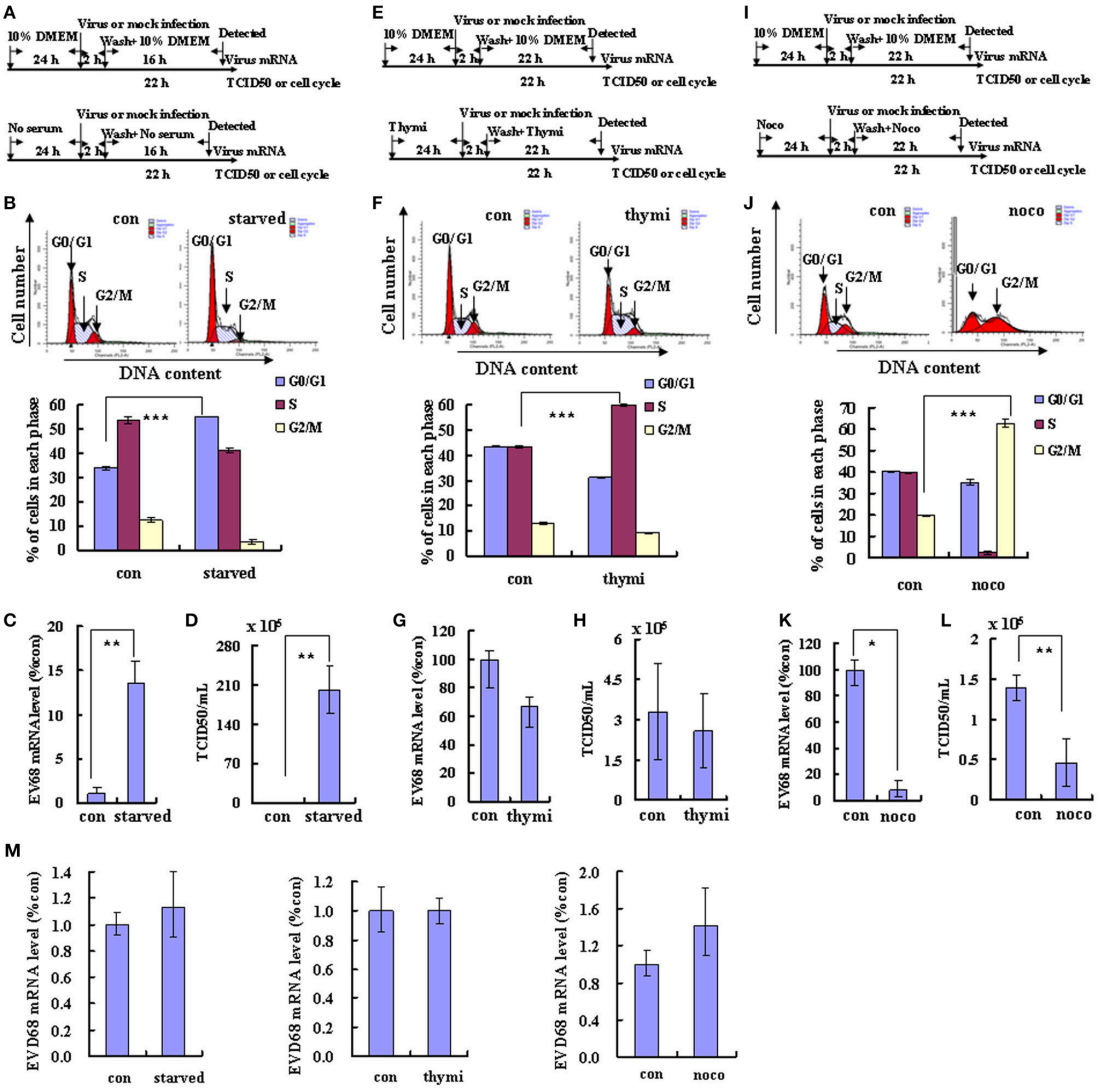
**Different cell cycle stages have profound effects on EV-D68 replication**. The effects of cell cycle synchronization on EV-D68 are shown for G0/G1 arrest **(A–D)**, S phase arrest **(E–H)**, and G2/M arrest **(I–L)**. **(A,E,I)** Flow diagram of how RD cells were treated with serum starvation (starved) for G0/G1 synchronization **(A)**, with thymidine (thymi) for S synchronization **(E)**, or with nocodazole (noco) for G2/M synchronization **(I)**. The top diagram in each panel shows the strategy for the control group, and the bottom panel shows the strategy for cell cycle synchronization. **(B,F,J)** Cell-cycle profiles were determined by flow cytometry after G0/G1, S, and G2/M synchronization with serum starvation, thymidine, and nocodazole treatment, respectively. Histograms below show the percentage of cells in each phase of the cell cycle as analyzed by the ModFit LT program. **(C,G,K)** Levels of intracellular EV-D68 Fermon strain RNA were detected in RD cells after cell cycle synchronization by quantitative real-time PCR. The results were standardized to GAPDH mRNA expression and normalized to 1.0 in mock-infected cells. **(D,H,I)** Progeny viruses in the supernatants were titrated using RD cells. A relative quantitative analysis of the TCID50/mL is shown. **(M)** Intracellular EV-D68 Fermon strain RNA levels were detected in RD cells with different cell cycle synchronization treatment by quantitative real-time PCR at post-infection 2 h. The results were standardized using GAPDH mRNA as a control and normalized to 1.0 in mock-infected cells. The results represent the mean ± S.D of three independent experiments. ^*^P < 0.05, ^**^P < 0.01, and ^***^P < 0.001.

Viral replication often is integrally associated with the cell cycle status of host cells (Feuer et al., 2002). To explore the possible benefits of different cell cycle phases for EV-D68 viral replication, we synchronized cells in different phases and then assessed viral replication and virulence. First, we assessed the effects of G0/G1 synchronization by serum deprivation (Darzynkiewicz et al., 1980). RD cells were cultured in either serum medium (control) or serum-free medium (G0/G1 synchronization) for 24 h. Then the cells were infected with the same titer of 0.8 MOI of EV-D68 (Fermon strain) or were mock-infected for 2 h, and either serum medium or serum-free medium was added for another 24 h (Figure 1A). As previously reported (He et al., 2010), serum deprivation induced obvious G0/G1 arrest as assessed by flow cytometry (*P* < 0.001; Figure 1B). At 2 h post-infection (viral entry stage), the EV-D68 genomic RNA levels were not significantly different in the control and serum-starved cells (Figure 1M); however, at 18 h post infection (viral replication stage) 13.55 times more viral RNA was detected in the serum-starved cells than in the control cells (*P* < 0.01; Figure 1C). Furthermore, at 24 h (viral production stage) the TCID50/mL of infectious EV-D68 particles was 348.84 times higher for supernatant from G0/G1 phase-synchronized cells (202.17 ± 42.60 × 10^5^) than for supernatant from control cells (0.59 ± 0.08 × 10^5^) (*P* < 0.01; Figure 1D). These results suggest that G0/G1-phase arrest does not affect viral entry, but promotes EV-D68 viral replication and production.

To determine whether viral replication and production also is elevated at other phases of the cell cycle, the effect of S phase synchronization was assessed. The cells were cultured in medium or were synchronized in S phase by culture with 0.85 mM thymidine for 24 h. Then, the cells were mock infected or were infected with 0.8 MOI of EV-D68 for 2 h, and fresh culture medium or 0.85 mM thymidine was added for another 24 h (Figure 1E). Thymidine induced obvious S phase arrest (P < 0.001; Figure 1F). The genomic RNA level remained similar in S phase-synchronized cells and control non-synchronized cells at 2 h post-infection (Figure 1M) and at 24 h post-infection (P > 0.05; Figure 1G). Furthermore, the TCID50/mL values at 24 h post-infection were equivalent for the S phase-synchronized cell supernatant (2.59 ± 1.37 × 10^5^) and the control cell supernatant (3.28 ± 1.80 × 10^5^) (*P* > 0.05; Figure 1H). These results suggest that S-phase arrest does not affect EV-D68 viral entry, replication or production.

To assess the effects of G2/M phase synchronization, cells were cultured in medium or were treated with 25 ng/ml nocodazole for 24 h; then, the cells were mock infected or were infected with EV-D68 at 0.8 MOI for 2 h, and cultured in fresh medium or 25 ng/ml nocodazole for another 24 h (Figure 1I). Nododazole induced obvious G2/M arrest (*P* < 0.001; Figure 1J). At 2 h post-infection, there was no significant difference in the genomic RNA level in the control and G2/M phase-synchronized cells (Figure 1M); however, at 24 h post-infection the genomic level was lower in the synchronized cells than in the control cells (*P* < 0.05; Figure 1K). Furthermore, at 24 h post-infection the TCID50/mL for supernatant from the G2/M phase-synchronized cells (0.46 ± 0.29 × 10^5^) was obviously lower than that from the control cells (1.40 ± 0.16 × 10^5^) (*P* < 0.01; Figure 1L). Therefore, these results suggest that G2/M synchronization does not affect viral entry, but inhibits EV-D68 viral replication and production.

### EV-D68 infection manipulates the host cell cycle and arrests cells at G0/G1

**Figure 2:**
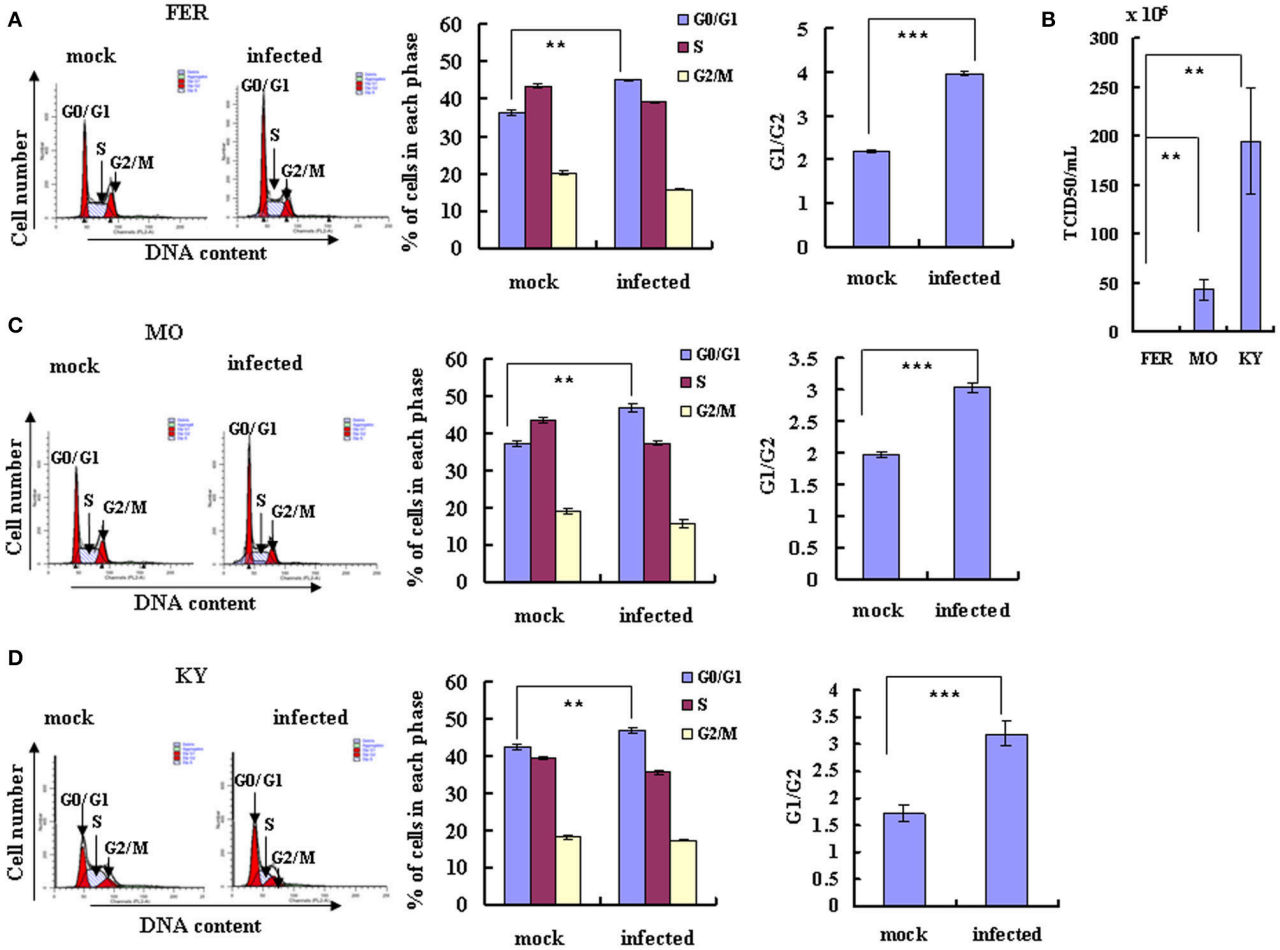
**EV-D68 infection induces G0/G1 arrest**. **(A,C,D)** RD cells were infected with EV-D68 Fermon **(A)**, US/MO/14-18947 **(C)** or US/KY/14-18953 **(D)** at an MOI of 0.8 or mock for 24 h for analysis of cell cycle status. Left: Cell-cycle profiles were determined by flow cytometry. Middle: The histograms show the percentage of cells in each phase of the cell cycle in mock-infected and EV-D68-infected cells. Right: The ratio of G0/G1 to G2/M is shown. **(B)** The virulence of Fermon, US/KY/14-18953 and US/MO/14-18947strains in infected RD cells was assessed 24 h post-infection with virus at MOI 0.8. Values are expressed as the TCID50/mL of the progeny viruses in the supernatant. The results represent the mean ± S.D of three independent experiments. ^**^P < 0.01 and ^***^P < 0.001.

Given that EV-D68 replication and production is dependent on the cell cycle, we next asked whether EV-D68 might have the ability to manipulate the host cell cycle to facilitate its own production. RD cells were infected with EV-D68 Fermon stain at an MOI of 0.8, and the cells were collected for cell cycle distribution analysis after 24 h. An obvious increase in the percentage of cells in G0/G1 was observed in EV-D68-infected cells (45.20 ± 0.14%) as compared to mock-infected cells (36.50 ± 0.76%) (23.84% increase; *P* < 0.01; Figure 2A). Therefore, EV-D68 itself can manipulate the host cell to accumulate preferentially at G0/G1 phase rather than at G2/M, which favors viral production.

Though the latter experiments were performed using the Fermon strain of EV-D68, which was isolated from 4 children with pneumonia and bronchiolitis in the United States in 1962 (Schieble et al., 1967; Zhang et al., 2015), several more recent strains of the virus have been isolated. The currently circulating EV-D68 US/MO/14-18947 and US/KY/14-18953 strains are similar to the Fermon strain in clinical characteristics and genome structure, but it is not known whether they are similar in virulence and ability to manipulate the cell cycle. Therefore, we compared the US/MO/14-18947 and US/KY/14-18953 strains to the Fermon strain. Under normal culture conditions, RD cells were infected with three strains virus at an MOI of 0.8 for 24 h, respectively, the TCID50/mL of the US/MO/14-18947 strain (43.07 ± 10.22 × 10^5^) was 29.76 times higher and the TCID50/mL of the US/KY/14-18953 (195.00 ± 54.03 × 10^5^) was 138.29 higher than the TCID50/mL of the Fermon strain (1.40 ± 0.16 × 10^5^) (Figure 2B). These results suggest that the EV-D68 virulence has increased over time, which could explain the recent rise in the incidence of Enterovirus-related disease (Esposito et al., 2015; Farrell et al., 2015; Reiche et al., 2015; Zhang et al., 2015).

Next, we assessed the cell cycle distribution after RD cells were infected with the currently circulating strains of EV-D68 at an MOI of 0.8 for 24 h. An obvious increase in the percentage of cells in G0/G1 phase was observed for both US/MO/14-18947 (25.83% increase; *P* < 0.01) (Figure 2C) and US/KY/14-18953 (10.85% increase; *P* < 0.01) (Figure 2D). These strains also caused a corresponding increase in the ratio of cells in G0/G1–G2/M (53.54% increase for US/MO/14-18947; 85.91% increase US/KY/14-18953; *P* < 0.001; Figures 2C,D). Therefore, the currently circulating strains possess increased virulence and a similar ability as the Fermon strain to skew the cell cycle toward the G0/G1 phase, which facilitates viral production.

### EV-D68 infection inhibits G0/G1 exit

**Figure 3:**
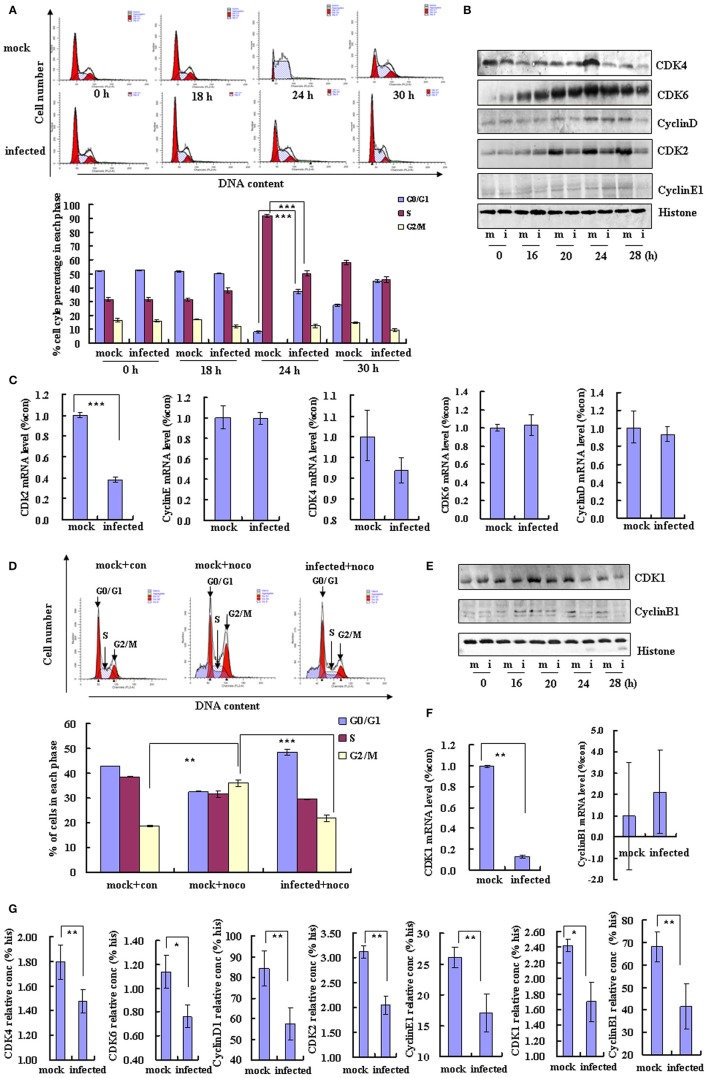
**EV-D68 infection prevents cell exit from G0/G1 into S phase and promotes G2/M to G0/G1 transition**. **(A)** RD cells were serum-starved for 24 h and then mock-infected (mock) or infected with EV-D68 Fermon strain (infected) at an MOI of 0.8. After 2 h of virus adsorption, the cells were treated with medium without FBS for 18 h, followed by medium containing 10% FBS. Top panel: The cell cycle profiles were determined by flow cytometry at 0, 18, 24, and 30 h post-infection. Bottom panel: The histograms show the percentage of cells in each phase of the cell cycle. **(B)** G0/G1 and S phase-related cell cycle proteins were analyzed by western blot analysis. RD cells were mock-infected (m) or infected with EV-D68 Fermon strain at an MOI of 0.8 (i) for 0, 16, 20, 24, and 28 h. Histone is shown as a loading control. Results are representative of three independent experiments. **(C)** At 24 h post-infection, mRNA levels of CDK2, cyclinE1, CDK4, CDK6, and cyclinD were evaluated in mock infected (mock) and EV-D68 infected (infected) cells by quantitative real-time PCR. The results are standardized to GAPDH and normalized to 1.0 in mock-infected cells. **(D)** RD cells were treated with 25 ng/mL nocodazole or control medium for 24 h, infected with EV-D68 Fermon strain at an MOI of 0.8 or mock for 2 h and then re-treated with 25 ng/mL nocodazole for synchronization. Top panel: Cell-cycle profiles were determined by flow cytometry. Low panel: The histograms show the percentage of cells in each phase of the cell cycle. **(E)** G2/M phase-related proteins were detected by western blot analysis. Results are representative of three independent experiments. **(F)** At 24 h post-infection, mRNA levels of CDK1 and cyclinB1 were assessed in mock-infected (mock) and EV-D68 infected (infected) cells by quantitative real-time PCR. The results are standardized to GAPDH mRNA and normalized to 1.0 in mock-infected cells. **(G)** At 28 h post-infection, the protein concentration of CDK4, CDK6, cyclinD1, CDK2, cyclinE1, CDK1, cyclinB1, and histone were assessed in mock-infected (mock) and EV-D68 infected (infected) cells by Elisa kit. The results are standardized to Histone (his). The results indicate the mean ± S.D of three independent experiments. ^*^P < 0.05, ^**^P < 0.01, and ^***^P < 0.001.

To further understand the mechanism of EV-D68 manipulation of the host cell cycle, we assessed whether EV-D68 could regulate cell cycle exit from G0/G1 into S phase. RD cells were synchronized in G0/G1 by serum starvation for 24 h, and then mock-infected or infected with EV-D68 Fermon strain for 2 h. The cells were then stimulated with 10% FBS in order to trigger cell cycle re-entry into S phase from G0/G1. At 24 h of mitogenic stimulation with serum, the mock-infected cells progressed synchronously from G0/G1 into S phase. In contrast, the majority of the EV-D68-infected RD cells remained in G0/G1 phase over the 24 h time period without S entry (*P* < 0.001; Figure 3A). Therefore, these results support a model in which EV-D68 infection regulates the cell cycle by preventing entry into the S phase.

Cyclin/CDK complexes are known to regulate cell cycle progression (Sherr, 1994). To identify the key molecules and signaling pathways that may mediate the inhibition of cell entry into S phase by EV-D68, we examined the protein expression profiles of host G0/G1-phase and S-phase proteins by Western blotting of RD cells at 0, 16, 20, 24, and 28 h post-infection. Among the molecules CDK4, CDK6, and cyclinD (which mediate cell cycle progression in G0/G1; Massagué, 2004) and CDK2 and cyclinE1 (which mediate cell cycle transition from G0/G1 to S phase; Hinds et al., 1992), the expression of CDK6 was not changed, and the expression of cyclinE1 was increased at 24 h post-infecion (Figure 3B), while all of them were significantly decreased in virus-infected cells as compared to mock-infected cells at 28 h post-infection (Figures 3B,G). Furthermore, the CDK2 mRNA level was decreased by EV-D68 infection; however, there were no significant differences between the virus and mock-infected groups in CDK4, CDK6, cyclinD or cyclinE1 mRNA levels (Figure 3C). Therefore, EV-D68 infection inhibits host expression of several cell cycle proteins, which is consistent with its ability to inhibit G0/G1 to S phase entry, and the modulation is likely to occur transcriptionally for CDK2 and post-transcriptionally for CDK4, CDK6, cyclinD, and cyclinE1.

### EV-D68 infection promotes G0/G1 entry

To further examine the potential effect of virus infection on cell cycle transition from G2/M phase into G0/G1, RD cells were treated with 25 ng/ml nocodazole or medium for 24 h for G2/M phase synchronization and then the cells were mock infected or infected with EV-D68 at 0.8 MOI for 2 h. Next, the cells were treated for an additional 24 h with 25 ng/ml nocodazole or fresh medium. Nocodazole induced obvious G2/M cell cycle arrest (35.91 ± 1.44 vs. 18.77 ± 0.20%; *P* < 0.01); however, after EV-D68 infection for 24 h, the percentage of cells in G2/M was decreased (21.82 ± 1.07 vs. 35.91 ± 1.44; *P* < 0.001), and the percentage of cells in G0/G1 was increased (48.59 ± 1.22 vs. 32.51 ± 0.21; *P* < 0.01) (Figure 3D). Therefore, EV-D68 infection also regulates the cell cycle by promoting exit from G2/M phase. Consistent with these findings, the expression of cyclinB1 and CDK1 (which mediate G2/M progression; Coverley et al., 2002; Yam et al., 2002) was down regulated by EV-D68 infection (Figures 3E,G). CDK1 was decreased at the mRNA level upon EV-D68 infection (*transition fromP* < 0.01), but cyclinB1 mRNA expression was not significantly regulated (Figure 3F). Therefore, EV-D68 virus promotes cell cycle exit from G2/M and entry into G0/G1 by modifying the pathway of G0/G1 entry at the transcriptional level for CDK1 and at the post-translational level for cyclinB1.

### The non-structural proteins 3D and 3C of EV-D68 mediate cell cycle alterations

**Figure 4:**
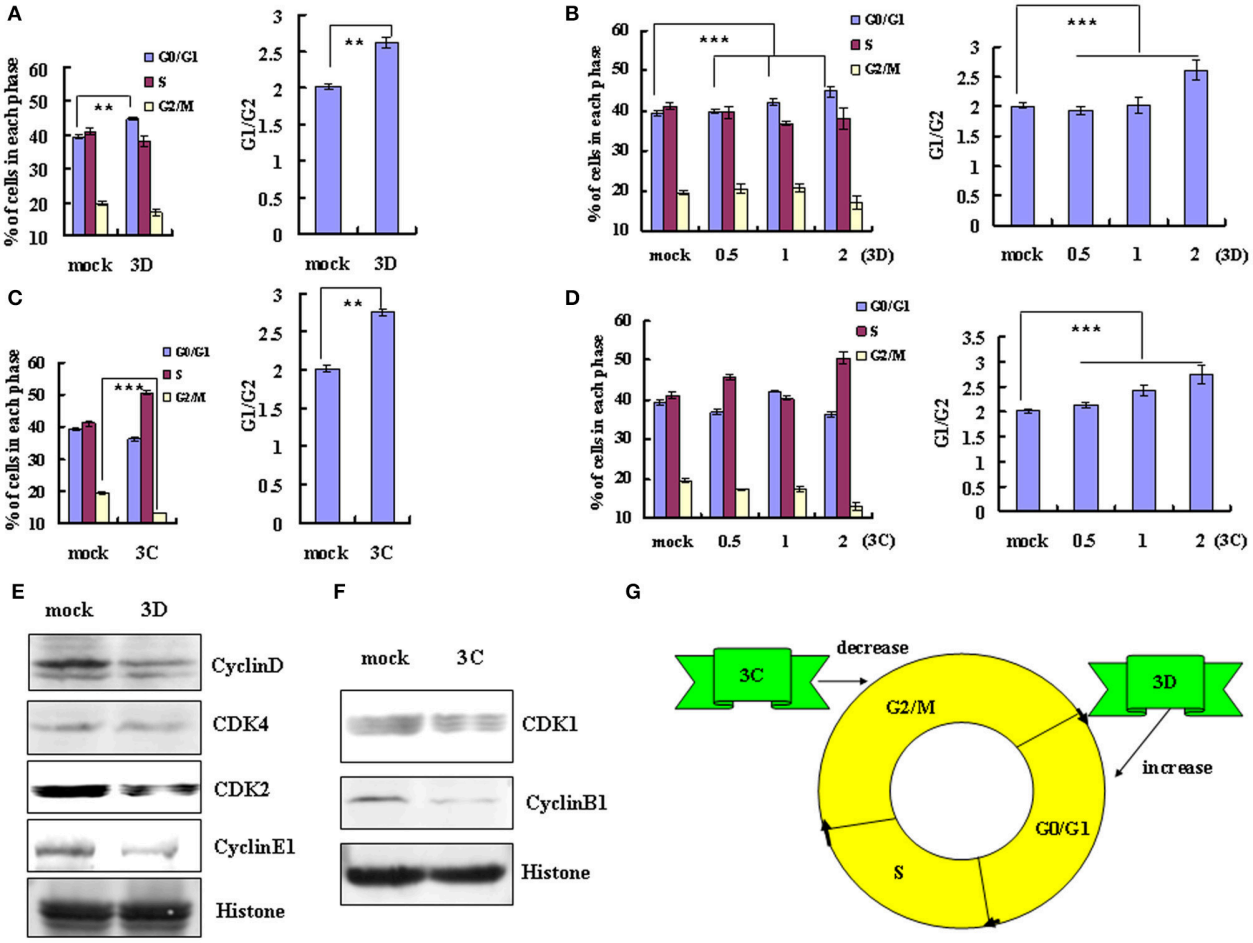
**The non-structural protein 3D and 3C of EV-D68 mediates cell cycle alterations. (A,C)** Effect of 3D and 3C on cell cycle progression. Left panel: The distribution of cell cycle in 293T cells was analyzed at 36 h after transfection with 2 μg of VR1012-3D-HA (3D), VR1012-3C-HA (3C) or the corresponding control vector VR1012 (mock). Right panel: The ratio of cells in G0/G1 to G2/M at 36 h after transfection with 2 μg of VR1012-3D-HA (3D), VR1012-3C-HA (3C) or the corresponding control vector VR1012 (mock). **(B,D)** The cell cycle distribution in 293T cells was analyzed at 36 h after transfection with 0, 0.5, 1, or 2 μg plasmid as indicated. Left panel: The histograms show the percentage of each phase in the cell cycle. Right panel: The ratios of cells in G0/G1 to G2/M. **(E,F)** The expression of cell cycle-related proteins after transfection of 293T cells with 3D, 3C or corresponding vector (mock) was assessed by Western blot analysis at 36 h. Histone is shown as a loading control. **(G)** Model for the distinct effects of the non-structural proteins 3D and 3C in cell cycle arrest caused by EV-D68. The results indicate the mean ± S.D of three independent experiments. ^**^P < 0.01 and ^***^P < 0.001.

A previous study concluded that exogenous expression of EV-A71 viral non-structural 3D protein, an RNA-dependent RNA polymerase, mediates cell cycle arrest at S phase (Yu et al.). Given this finding, we examined whether non-structural 3D protein of EV-D68 had the same ability to mediate cell cycle alteration. Transfection of 3D expression vector (2 μg) induced G0/G1 arrest, with an increase in the percentage of G0/G1 cells from 39.37 ± 0.52% to 44.76 ± 1.29% (13.69% increase; *P* < 0.01) and a corresponding increase in the G0/G1–G2/M ratio from 2.02 ± 0.04 to 2.62 ± 0.18 (29.70% increase; *P* < 0.01) (Figure 4A). Furthermore, the extent of the increase in the percentage of G0/G1 cells was dependent on the dose of 3D vector (0, 0.5, 1, 2 μg; *R* = 0.932; *P* < 0.001), and the G0/G1 to G2/M ratio also depended on the dose of 3D vector (*R* = 0.827; *P* < 0.001; Figure 4B). These results suggest that the non-structural protein 3D of EV-D68 contributes to G0/G1 arrest.

We also examined the potential role of non-structural protein 3C in cell cycle regulation by EV-D68. Transfection of high dose 3C expression vector (2μg) decreased the percentage of cells in G2/M phase from 19.53 ± 0.26% to 13.19 ± 0.48% (32.46% decrease) and increased in the ratio of cells in G0/G1 to G2/M from 2.01 ± 0.04 to 2.75 ± 0.17 (36.82% increase; *P* < 0.01) (Figure 4C). High dose 3C transfection also increased the percentage of cells in S phase (from 41.10 ± 0.48% to 50.53 ± 0.73%). Although the change in the cell cycle profile was not dose-dependent, the G0/G1 to G2/M ratio was dose-dependent (*R* = 0.951; *P* < 0.001; Figure 4D). These results suggest that the non-structural protein 3C may contribute to the enhanced cell cycle exit from G2/M phase after EV-D68 infection.

To verify these findings and to evaluate the mechanism of cell cycle regulation after 3D and 3C transfection, we performed Western blotting assays to examine their effect on the expression of cycle-related proteins. Consistent with the cell cycle analyses, 3D down-regulated the expression of cyclinD, CDK4, CDK2, and cyclinE1 (Figure 4E), while 3C down-regulated the expression of CDK1 and cyclinB1 (Figure 4F). Therefore the non-structural protein 3C facilitates exit from G2/M and the non-structural protein 3D mediates arrest in G0/G1 (Figure 4G).

### G0/G1-phase synchronization has distinct effects on EV-D68 and EV-A71 viral replication

**Figure 5:**
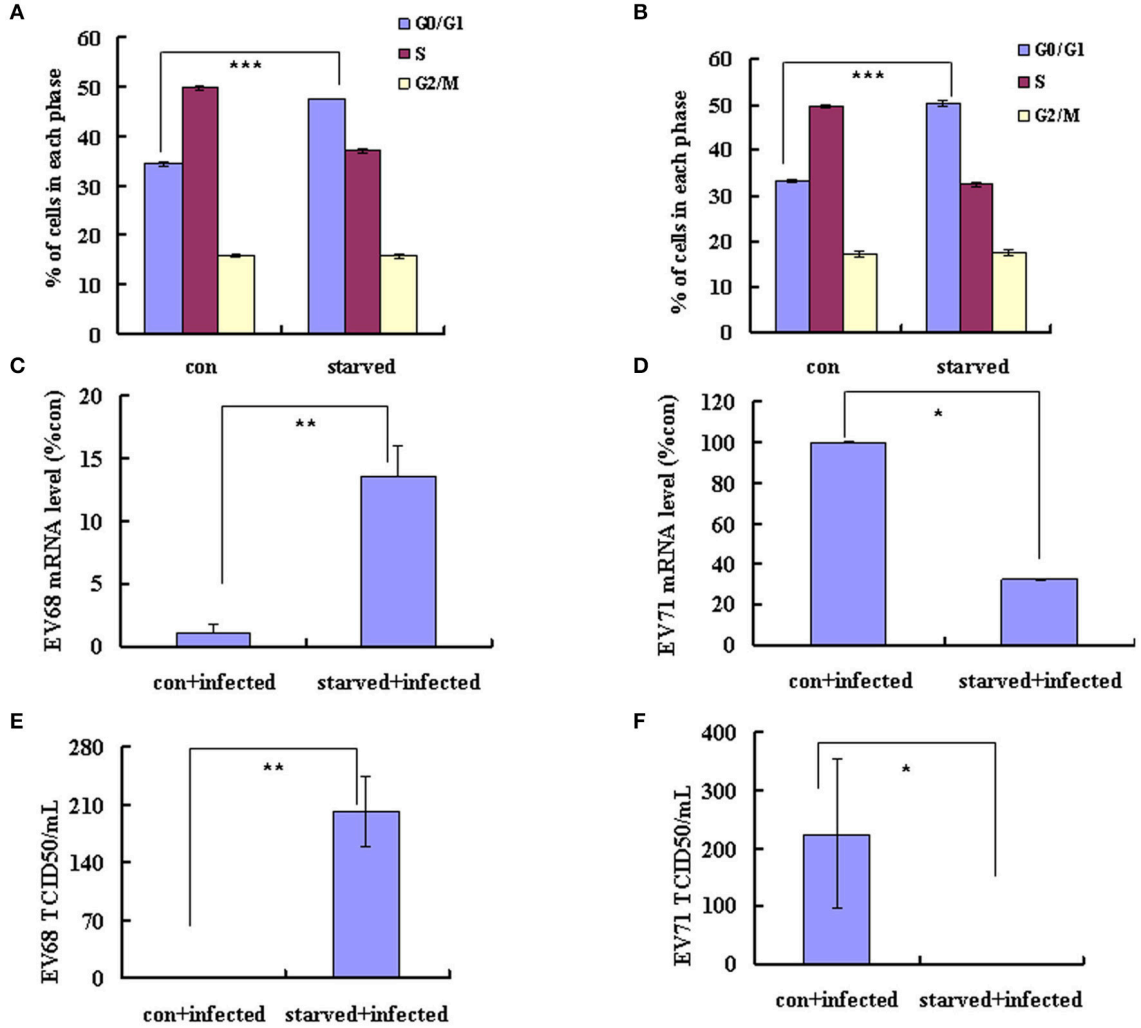
**G0/G1-phase synchronization has different effect on the replication of EV-A71 and EV-D68**. RD cells infected with EV-D68 Fermon strain **(A,C,E)** or EV-A71 **(B,D,F)** at an MOI of 0.8 and the effects of synchronization by starvation were assessed. **(A,B)** RD cells were serum-starved for 48 h to synchronize cells in G0/G1 phase. The cell-cycle distribution was then detected by flow cytometry. The histograms showed the percentage of each phase in the cell cycle. **(C,D)** At 18 h post-infection, intracellular EV-D68 and EV-A71 RNA levels were assessed in control medium (con+infected)-treated or no serum (starved+infected)-treated RD cells by quantitative real-time PCR. The results were standardized using GAPDH mRNA as a control and normalized to 1.0 in mock-infected cells. **(E,F)** At 24 h post-infection, The TCID50/mL was shown through titrating the progeny viruses of EV-D68 or EV-A71 in the supernatants with RD cells. The results represent the mean ± S.D of three independent experiments. ^*^P < 0.05, ^**^P < 0.01, and ^***^P < 0.001.

EV-D68 (serotype HEV-D) and EV-A71 (serotype HEV-A) are both enteroviruses. We have demonstrated that G0/G1 synchronization promotes EV-D68 viral replication (Figures 1A–D). However, in our previous study, we determined that G0/G1 synchronization inhibits EV-A71 viral replication. To exclude experimental variation as an explanation for the disparate responses of these viruses to G0/G1 synchronization, we performed a side-by-side comparison of the two enteroviruses. Our results confirm that no serum treatment induces G0/G1 synchronization (Figures 5A,B) and G0/G1 synchronization has opposite effects for the two viruses (Figure 5). After 18 h infection with EV-D68, the viral genomic mRNA level was 13.55 times higher in serum-starved cells than in control cells (*P* < 0.01; Figure 5C); however, after 18 h infection with EV-A71, the viral genomic mRNA level was 3.12 times lower in serum-starved cells (*P* < 0.05; Figure 5D). Furthermore, after 24 h infection, the TCID50/mL for EVD68 was 341.66 times higher for G0/G1 phase-synchronized cells than for control cells (202.17 ± 42.60 × 10^5^ vs. 0.59 ± 0.08 × 10^5^; *P* < 0.01; Figure 5E), while the TCID50/mL for EV-A71 was 489.37 times lower for G0/G1 phase-synchronized cells than for control cells (0.46 ± 0.15 × 10^5^ vs. 225.11 ± 129.36 × 10^5^; *P* < 0.05; Figure 5F). These results confirm that G0/G1-phase arrest has different effects for the two enteroviruses.

### G2/M-phase synchronization has similar effects on different strains of EV-D68 and EV-A71

**Figure 6:**
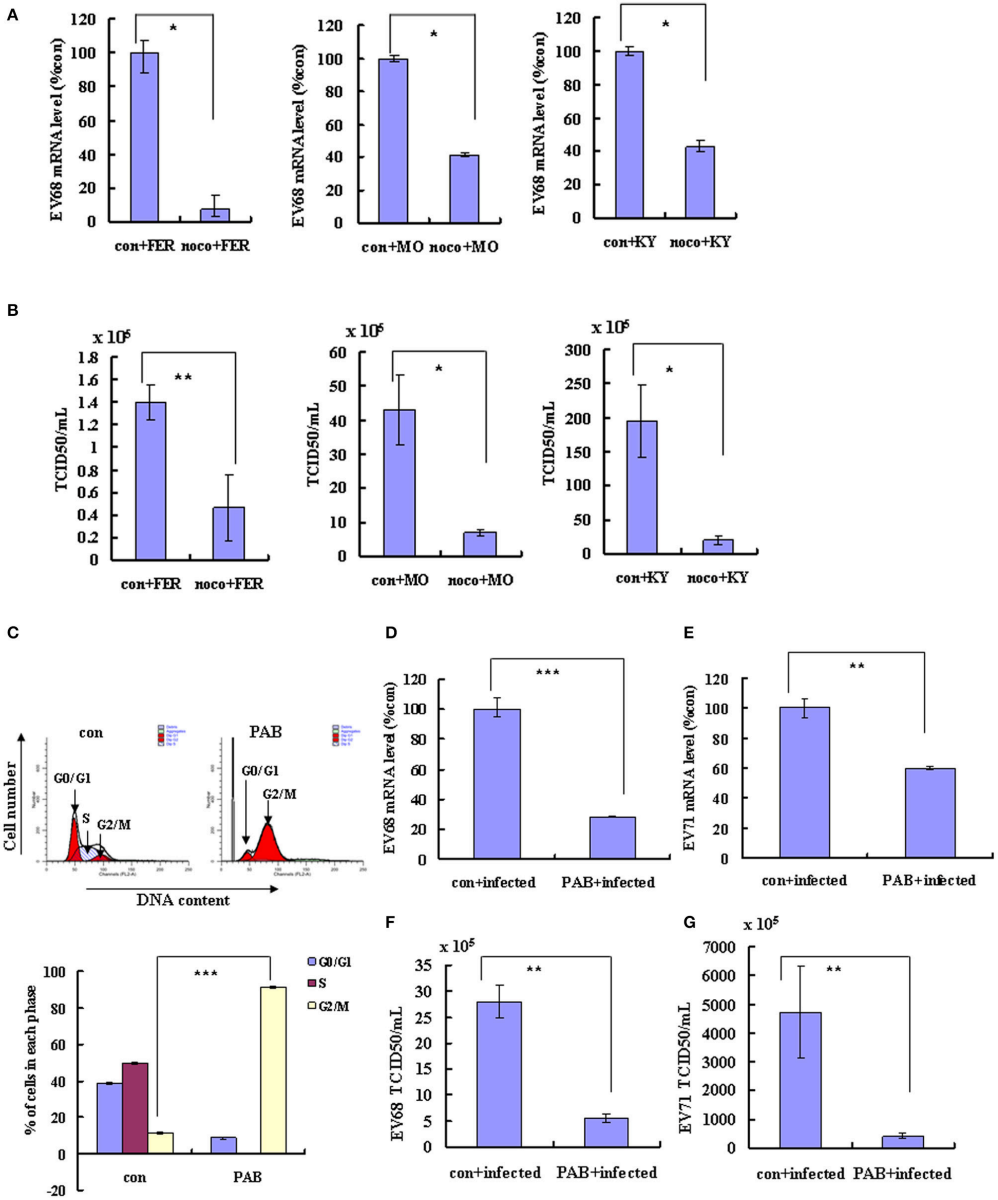
**Synchronization in the G2/M phase inhibits the replication of EV-D68 and SV-A71**. **(A–C)** RD cells were treated with or without 25 ng/mL nocodazole (noco) for 24 h, infected with EV-D68 Fermon (FER), US/MO/14-18947 (MO) or US/KY/14-18953 (KY) strains at an MOI of 0.8 for 2 h, and then treated again with or without 25 ng/mL nocodazole for synchronization. **(A)** At 24 h post-infection, intracellular EV-D68 RNA levels were detected by quantitative real-time PCR. The results were standardized using GAPDH mRNA as a control and normalized to 1.0 in mock-infected cells. **(B)** At 24 h post-infection, the TCID50/ml of the progeny viruses was determined. **(C–G)** RD cells were infected with EV-D68 strains US/KY/14-18953 or EV-A71 at an MOI of 0.8 for 2 h, and then treated with 2 μM Pseudolaric acid B (PAB) for 24 h for G2/M synchronization. **(C)** RD cells were treated with 2 μM Pseudolaric acid B (PAB) for 24 h for G2/M synchronization. Top panel: Cell-cycle profiles were determined by flow cytometry. Bottom panel: The histograms show the percentage of cells in each phase of the cell cycle. **(D,E)** Intracellular EV-D68 or EV-A71 RNA levels were assessed in control medium (con+infected) or PAB containing medium (PAB+infected)-treated RD cells by real-time quantitative PCR. The results are standardized by GAPDH mRNA as a control and normalized to 1.0 in control-infected cells. **(F,G)** The progeny viruses of EV-D68 or EV-A71 in the supernatant were titrated using RD cells, and the TCID50/mL was shown. The results indicate the mean ± S.D of three independent experiments. ^*^P < 0.05, ^**^P < 0.01, and ^***^P < 0.001.

G2/M synchronization with nocodazole has been shown to inhibit both the EV-D68 Fermon strain in this study (Figures 1I–L) and EV-A71 in our previous study (Yu et al., 2015). To determine whether similar effects of G2/M-phase synchronization are observed for the currently circulating strains of EV-D68, cells were treated with 25 ng/ml nocodazole or medium for 24 h, and were then infected at 0.8 MOI for 2 h and treated with 25 ng/ml nocodazole or fresh medium for another 24 h. Our results demonstrate that nocodazole treatment decreased the genomic RNA levels (Figure 6A) and the TCID50/ml value (Figure 6B) of Fermon, US/KY/14-18953 and US/MO/14-18947, which suggests that the virus inhibition upon G2/M synchronization may be similar for all enteroviruses.

To confirm that G2/M synchronization inhibits EV-D68 and EV-A71, we assessed the effects of an alternate agent that can exert G2/M arrest *in vitro*, pseudolaric acid B (PAB), which is a diterpene acid isolated from the root and trunk bark of *Pseudolarix kaempferi Grord (Pinaceae)* (Yu et al., 2007, 2013). PAB was confirmed to promote G2/M arrest after 24 h (Figure 6C). To assess the effects of PAB on viral RNA and virulence, RD cells were infected with EV-D68 US/KY/14-18953 strain or EV-A71 at an MOI of 0.8 for 2 h and then treated with 2 μM PAB for 24 h. PAB decreased the genomic RNA levels of both EV-D68 (28.32% of control; P < 0.001; Figure 6D) and EV-A71 (59.87% of control; P < 0.001; Figure 6E). Furthermore, PAB decreased the TCID50/ml value of both EV-D68 (80.31% decrease; *P* < 0.01; Figure 6F) and EV-A71 (91.27% decrease; *P* < 0.01; Figure 6G). These results confirm that G2/M arrest inhibits both the EV-D68 and EV-A71 strains, which suggest a common approach for therapeutic intervention that might potentially target a broader range of enteroviruses.

## Discussion

Enterovirus 68 (EV-D68) usually causes mild to severe respiratory illness, including runny nose, sneezing, cough, body and muscle ache, wheezing, difficulty breathing, and in the cases of some infants, children and teens, death. Although EV-D68 was first identified in California in 1962, the number of people in one breakout in 2014 with confirmed EV-D68 infection was much greater than the number reported in previous years (http://www.cdc.gov/non-polio-enterovirus/about/ev-d68.html). It is hard to predict whether EV-D68 will emerge again in future outbreaks, but the value of resolving the pathogenic mechanism of EV-D68 is obvious. In this study, we investigated the pathogenic mechanism of EV-D68 to reveal the relationship between virus infection and the host cell cycle.

To assess the possibility that the cell cycle status affects EV-D68 viral replication, we first synchronized cells in G0/G1. Our results demonstrate that G0/G1 arrest promotes EV-D68 replication and increases viral virulence without affecting virus entry. We also assessed the effects of S phase and G2/M phase synchronization on viral production. Our results suggest that S phase synchronization does not affect viral entry, replication or production compared to the control treatment, while G2/M synchronization inhibits viral replication and decreases viral virulence, but does not affect virus entry. These results indicate that G0/G1 phase is most favorable for EV-D68 replication, that S phase can support some viral production, and that G2/M phase is inhibitory for host viral production.

Given that G0/G1 phase supports EV-D68 production, it would be advantageous for the virus to manipulate the host cell cycle to increase viral production. Indeed, the EV-D68 Fermon strain displayed significant ability to increase the percentage of cells in G0/G1 phase. The EV-D68 Fermon strain was isolated in the United States in 1962 (Schieble et al., 1967), but currently circulating strains, including EV-D68 US/MO/14-18947 and US/KY/14-18953, may be more relevant to current human health. Therefore, we examined whether these two currently circulating strains had similar ability to manipulate the cell cycle. Our results confirmed that the circulating EV-D68 strains manipulate the cell cycle in a similar manner as does the Fermon strain, though the current strains have higher virulence than Fermon. Therefore, after more than 50 years' evolution, EV-D68 still possesses the ability to arrest cells at G0/G1 phase but EV-D68 virulence has increased.

To pinpoint the cause for G0/G1 accumulation upon EV-D68 infection, we analyzed the transitions from G0/G1 into S and from G2/M into G0/G1. After cell cycle release from G0/G1, mock-infected cells entered S phase, while EV-D68-infected cells still accumulated at G0/G1 phase, thus demonstrating that EV-D68 infection prevents S phase entry from G0/G1 phase. Furthermore, after G2/M synchronization, EV-D68 infection promoted cell cycle transition from G2/M into G0/G1 phase. Therefore, EV-D68 infection regulates G0/G1 cell cycle arrest both by promoting G0/G1 phase entry and by inhibiting G0/G1 phase departure. To further analyze the mechanism of host cell cycle manipulation by EV-D68, we assessed the expression of cyclins and CDKs that are known to form complexes to regulate cell cycle progression (Oosthuysen et al., 2015). For example: cyclinD/CDK4 and cyclinD/CDK6 regulate G0/G1 progression (Massagué, 2004); cyclinE/CDK2 regulates S-phase entry from G1 (Hinds et al., 1992); cyclinA/CDK2 regulates S-phase progression by replacing cyclinE (Coverley et al., 2002; Yam et al., 2002); and cyclinB1/CDK1 prevents cell cycle transition from G2/M into G0/G1 (Yu et al., 2013; Adeyemi and Pintel, 2014). We demonstrated that cyclinD, CDK4, CDK6, cyclinE1 and CDK2 are down-regulated after EV-D68 infection, which could explain the ability of EV-D68 to inhibit the transition from G0/G1 to S phase. Furthermore, the expression of cyclinB1 and CDK1 was down-regulated after EV-D68 infection, which is consistent with the ability of EV-D68 to promoting the transition from G2/M to G0/G1. Therefore, expression of cell cycle-related proteins further supports our results suggesting that EV-D68 induces G0/G1 arrest by regulating G0/G1 phase entry and exit. We also analyzed whether the regulation of protein expression occurred at the mRNA level and found that CDK2 and CDK1 were down-regulated by EV-D68, but that the other cell cycle proteins were not. This indicates that the regulation of cellular factors related to the cell cycle by EV-D68 occurs partly at the transcriptional level, but mostly occurs at the post-translational level.

Viral non-structural proteins are often essential for viral replication, so we also evaluated whether EV-D68 might exert its host cell cycle regulatory function via its viral non-structural proteins. Our results confirmed that the non-structural protein 3D increases the percentage of cells in G0/G1 phase by decreasing the expression of the G0/G1 and S phase related-cell cycle proteins cyclinD, CDK4 and cyclinE1, while 3C decreases the percentage of cells in G2/M phase by decreasing the expression of G2/M-related proteins CDK1 and cyclinB1. These findings raise the possibility that the non-structural proteins 3D and 3C may function coordinately in the context of an EV-D68 infection to enhance the percentage of cells in G0/G1 and increase the G0/G1 to G2/M ratio. It is noted that non-structural 3D protein of EV-A71 mediates cell cycle arrest at S phase, while 3D of EV-D68 mediated cell cycle at G0/G1, although until now the detailed reason of the difference is not clear, it is speculated that 290 of same amino acid sequence might bind with a same target which is responsible for the function of cell cycle arrest, while 172 of different amino acid sequence might bind other different factors which is responsible for different ability of cell cycle regulation, and this part will be investigated in the future.

In the current study, we demonstrated that G0/G1 phase arrest was required by EV-D68 production; however, in a previous study, EV-A71 was shown to have the opposite effect (Yu et al., 2015). This difference is surprising given that both EV-A71 and EV-D68 belong to the *Picornaviridae* family. To further confirm the different activities between the two viruses, we analyzed them side by side, and our results confirm that G0/G1 synchronization promotes EV-D68 viral production but inhibits EV-A71 viral production. Therefore, EV-D68 an EV-A71 can both manipulate the host cell cycle, but the process and outcomes of cell cycle manipulation are entirely different, which could explain why EV-D68 and EV-A71 have varying characteristics, such as the epidemic region (Europe and Asia), the clinical symptoms, and the epidemic size. Meanwhile, these results serve as a reminder that different enteroviruses may require different therapeutic treatments targeting a different set of host factors. Unfortunately, the reason of the difference between them is not clear, but it is speculated that some a host factor(s) generated or kept high level in G0/G1 phase was required by EV-D68 production, while EV-A71 required some a host factor(s) generated or kept high level in S phase so that their non-structural 3D were obligated by two viruses to regulate host cell cycle for their own replication.

The main goal of analyzing the mechanism of EV-D68 pathogenesis was to identify new strategies for preventing and treating the disease, so we did additional experiments to further explore G2/M synchronization as an approach to inhibit the replication of different EV-D68 strains. Our results demonstrate that in addition to its effects on the EV-D68 Fermon strain, G2/M synchronization by nocodazole inhibited the replication and virulence of US/KY/14-18953 and US/MO/14-18947. Additionally, PAB, which is another agent that can induce G2/M arrest, also significantly inhibited the production of EV-D68 and EV-A71. Because nocodazole also has been shown to be effective in inhibiting the production of EV-A71 (Yu et al., 2015), medicines that induce G2/M arrest might be considered as a common approach for inhibiting different types of anti-enterovirus infection, which provides a new direction for anti-enterovirus drug development.

## Ethics statement

This study has obtained ethics approval from the ethics committee at the First Hospital of Jilin University.

## Author contributions

JY, X-fangY, and SH designed the experiments and wrote the paper, ZW and JY conducted the experiments, TZ and YW analyzed cell culture, FS, X-fengY and WZ prepared the virus, LX prepared the reagents.

### Conflict of interest statement

The authors declare that the research was conducted in the absence of any commercial or financial relationships that could be construed as a potential conflict of interest.
